# Factors for postoperative recurrence of orbital solitary fibrous tumor: an analysis of long‐term clinical follow‐up results from a Chinese tertiary hospital

**DOI:** 10.1186/s12886-021-01825-6

**Published:** 2021-01-26

**Authors:** Peng Yang, Hao-Cheng Liu, E Qiu, Wei Wang, Jia-Liang Zhang, Li-Bin Jiang, Hong-Gang Liu, Jun Kang

**Affiliations:** 1 Department of Neurosurgery, Tongren Hospital of China Capital Medical University, Chongwenmennei Street, Dongcheng District, 100730 Beijing, China; 2 Department of Ophthalmology, Tongren Hospital of China Capital Medical University, 100730 Beijing, China; 3 Department of Pathology, Tongren Hospital of China Capital Medical University, 100730 Beijing, China

**Keywords:** Orbital, Transorbital, Transfronto‐orbital, Surgical approach, OSFT

## Abstract

**Background:**

This study analyzed the clinical features, imaging manifestations, histopathology, immunohistochemistry, and surgical approaches of the orbital solitary fibrous tumor (OSFT), as well as the factors for postoperative recurrence of such disease.

**Methods:**

The clinical data of 16 patients with OSFT treated in our center from 2003 to 2020 were analyzed retrospectively, and the clinical symptoms, treatment methods, and follow-up results were recorded.

**Results:**

Of the 16 patients, 8 were females (50.0 %) and 8 were males (50.0 %); the average age of treatment was 37 ± 7 years and the median follow-up time was 74 (8, 228) months. Sixteen patients with OSFT underwent a total of 29 operations, of which 12 were transorbital approach operations and 17 were transfronto-orbital approach operations. Ten patients (10/16, 62.5 %) had recurrence. The recurrence rate of transorbital approach operations was 83.3 % (10/12), and the recurrence rate of transfronto-orbital approach operations was 17.6 % (3/17). No patients had treatment-related complications.

**Conclusions:**

The main pathological feature of OSFT is a benign tumor. OSFT has a tendency to grow toward the cranio-orbital junction. The postoperative recurrence rate of OSFT is relatively high, so complete tumor resection is very important for prognosis. Inappropriate surgical approaches can lead to incomplete removal of the tumor and cause recurrence. Choosing the correct operation approach according to the position of the OSFT in the orbit and complete removal of the dura mater and bone affected by the tumor is crucial for the prognosis. Nevertheless, regular long-term follow-up after complete resection is necessary.

## Background

The solitary fibrous tumor (SFT) occurs most often in the pleura, other positions also could be found [1]. Solitary fibrous tumor of the orbit is a rare mesenchymal tumor in the soft tissue of the orbit. In recent years, several cases of OSFT had been reported, in 1994 Dorfman [2] and Westra [3] reported for the first time of the OSFT. Then more and more neurosurgeons and ophthalmologists recognized and reported such tumor [4–8], SFT tumor is extremely rare in the orbit. Intra-orbital OSFT is extremely rare, and cranio-orbital OSFT is even rarer [9–11]. There are a few related reports in many literatures. In recent years, OSFT has been reported in many pieces of literatures, but there are still many differences in tumor recurrence [12]. OSFT is not sensitive to radiotherapy and chemotherapy. Complete removal of the tumor determines the prognosis of the disease [13]. Most of the current surgical operations are performed by ophthalmologists [14–17], but the surgical approaches of ophthalmology are relatively limited. When the location of the tumor is not suitable for the operation of the ophthalmologist, an improper surgical approach may bring the possibility of tumor recurrence. The surgical approach of neurosurgery may provide better help for complete tumor resection. We reported the 16 cases of OSFT, with clinical features, imaging manifestations, histopathology, and immunohistochemistry, compared with different prognostic results brought by different surgical approaches.

## Materials and methods

### Subjects

This study retrospectively analyzed the clinical data of patients with OSFT treated in our center from January 2003 to October 2020. The researchers recorded clinical symptoms, treatment methods, CT, MRI, pathology specimens, and follow-up results. Imaging (CT, MRI) results were performed at each time point. The present study meets the requirements of the Declaration of Helsinki of the World Medical Association and has been approved by the Ethics Committee of our center. Patients or their family members provided informed consent.

### Inclusion and exclusion criteria

Inclusion criteria: (1) patients who were diagnozed as OSFT; (2) age was older than 18 years old; (3) patients who have signed informed consent. Exclusion criteria: (1) patients who had advanced malignant tumor; (2) patients whose datas were incomplete.

## Methods

According to the follow-up results, the location of the tumor in the orbit and the risk factors for recurrence were analyzed. The patients were all treated by surgical operations, the main surgical approaches were transorbital approach operation and transfronto-orbital approach operation. For tumors located in the superficial orbital region, the transorbital approach was used for resection; for patients with tumors located in the cranio-orbital region adjacent to the cavernous sinus invading the intracranial structure or recurring after transorbital approach operation, the transcranial approach operation would be adopted.

### Statistical analysis

We used the software program SPSS 20.0 (IBM, Chicago, USA) to conduct the statistical analysis. The continuous variables of normal distribution were expressed as mean ± standard deviation, the continuous variables of non-normal distribution were expressed as median (interquartile range [IQR]), the categorical variables were expressed as frequency (percentage[%]). A value of *P* < 0.05 was considered statistically significant.

## Results

### General characteristics

The study included 16 patients with OSFT, 8 patients were males and 8 patients were females. They were treated by two kinds of surgical operation, 29 operations in total. The average age of treatment was 37 ± 7 years, the median follow-up time was 74 (8, 228) months.

### Imaging characteristics

One OSFT was located in the extraconal space, fifteen in the retrobulbar intraconal space. Eleven OSFTs involved the cavernous sinus, superior orbital fissure area, and involved the endocranium. On T1-weighted images, all OSFTs seemed to be equally intensities to gray matter. On the T2-weighted image, the lesion showed anisotropic intensity in 15 patients and low intensity in 1 patient. Contrast-enhanced MRI showed that all lesions showed significantly enhanced heterogeneity. Fifteen OSFTs were accompanied by the proliferation, absorption, and destruction of orbital wall bone from CT scans.

### Histopathology and immunohistochemistry characteristics

All 16 patients received in our center underwent 29 operations, and all samples were diagnosed as OSFT by pathology. Surgical samples were collected for histological and immunohistochemical analyses. Immunohistochemical studies for CD34, CD99, Bcl-2, SMA, S-100 protein, Ki-67, CK, CD117 were tested in all samples. The CD34 was positive in all pathological samples (16/16, 100 %). The Ki-67 index of all 29 surgical samples was 5 % − 20 %. Besides, OSFTs has been showed to exhibit strong positivity with vimentin (12/16, 76 %), CD99 (9/16, 56.3 %), and Bcl-2 (11/16, 68.8 %). EMA (2/16, 12.5 %) and SMA (6/16, 37.5 %) are occasionally detected, while OSFT is usually rare for S-100 (1/16, 6.3 %) and CK (1/16, 6.3 %) expression.

### The follow‐up results

Sixteen patients completed follow up, the median follow-up time was 74 (8, 228) months. All patients with OSFT underwent a total of 29 operations, of which 12 were transorbital approach operations and 17 were transfronto-orbital approach operations. Ten patients (10/16, 62.5 %) had recurrence. The recurrence rate of transorbital approach operations was 83.3 % (10/12), and the recurrence rate of transfronto-orbital approach operations was 17.6 % (3/17). The overall recurrence rate of surgery was 44.8 % (13/29). No patients had treatment-related complications (Table 1).

**Table 1:** Surgical approaches and postoperative follow-ups of 16 cases of OSFTs

Patient No./Sex	Symptoms	Side	Treatment and Surgical Approach	Tumor location and involvement	Immuno-histochemistry		Progress	Prognosis	Follow-up time, m
					CD34	Ki-67			
1/F	Proptosis; Vision loss	R	Transorbital approach	Inside the muscle cone; lateral wall of the orbit;	+	+(<10 %)	Improvement of the proptosis and vision	Recurrence	120
	Proptosis; Vision loss	R	Transfronto-orbital approach	Inside the muscle cone; lateral wall of the orbit; cavernous sinus and superior orbital fissure	+	+(< 10 %)	Improvement of the proptosis and vision	None recurrence	92
2/F	Proptosis; Vision loss	L	Transorbital approach	Inside the muscle cone; lateral wall of the orbit;	+	+(< 10 %)	Improvement of the proptosis and vision	Recurrence	34
	Proptosis; Vision loss	L	Transorbital approach	Inside the muscle cone; lateral wall of the orbit;	+	+(< 10 %)	Improvement of the proptosis and vision	Recurrence	28
	Proptosis; Vision loss	L	Transfronto-orbital approach	Inside the muscle cone; lateral wall of the orbit; cavernous sinus and superior orbital fissure	+	+(< 10 %)	Improvement of the proptosis and vision	None recurrence	24
3/M	Proptosis; Visual field defect; Ocular movement disorder	L	Transorbital approach	Inside the muscle cone	+	+(< 10 %)	Improvement of the proptosis	Recurrence	18
	Proptosis; Visual field defect; Ocular movement disorder	L	Transfronto-orbital approach	Inside the muscle cone; lateral wall of the orbit;	+	+(< 10 %)	Improvement of the proptosis	None recurrence	24
4/M	Proptosis; Vision loss	R	Transorbital approach	Inside the muscle cone; lateral wall of the orbit	+	+(< 10 %)	Improvement of the proptosis	Recurrence	24
	Proptosis; Blindness	R	Transorbital approach	Inside the muscle cone; lateral wall of the orbit	+	+(< 10 %)	Orbital exenteration	Recurrence	8
	Cranio-orbital mass	R	Transfronto-orbital approach	Inside the muscle cone; lateral wall of the orbit; cavernous sinus and superior orbital fissure; Subdural brain tissue	+	+(< 20 %)	The mass disappeared	None recurrence	35
5/F	Proptosis	R	Transfronto-orbital approach	Inside the muscle cone; lateral wall of the orbit; cavernous sinus and superior orbital fissure	+	+(< 5 %)	Improvement of the proptosis	None recurrence	82
6/M	Proptosis	L	Transfronto-orbital approach	Inside the muscle cone; lateral wall of the orbit	+	+(< 5 %)	Improvement of the proptosis	Recurrence	228
	Proptosis; Blindness	L	Transfronto-orbital approach	Inside the muscle cone; lateral wall of the orbit; cavernous sinus and superior orbital fissure	+	+(< 5 %)	Improvement of the proptosis	None recurrence	31
7/F	Proptosis	R	Transorbital approach	Outside the muscle cone	+	+(< 10 %)	Improvement of the proptosis	None recurrence	30
8/F	Proptosis; Vision loss; Ocular movement disorder	R	Transorbital approach	Inside the muscle cone; lateral wall of the orbit	+	+(< 10 %)	Improvement of the proptosis	Recurrence	72
	Proptosis; Vision loss; Ocular movement disorder	R	Transorbital approach	Inside the muscle cone; lateral wall of the orbit	+	+(< 10 %)	Improvement of the proptosis	Recurrence	72
	Proptosis; Vision loss; Ocular movement disorder	R	Transfronto-orbital approach	Inside the muscle cone; lateral wall of the orbit; cavernous sinus and superior orbital fissure	+	+(< 10 %)	Improvement of the proptosis	None recurrence	53
9/M	Proptosis; Vision loss; Ocular movement disorder	L	Transfronto-orbital approach	Inside the muscle cone; lateral wall of the orbit; cavernous sinus and superior orbital fissure	+	+(< 10 %)	Improvement of the proptosis	Recurrence	54
	Proptosis; Vision loss; Ocular movement disorder	L	Transfronto-orbital approach	Inside the muscle cone; lateral wall of the orbit; cavernous sinus and superior orbital fissure	+	+(< 10 %)	Blindness	Recurrence	45
10/F	Proptosis; Vision loss;	L	Transfronto-orbital approach	Inside the muscle cone; lateral wall of the orbit; cavernous sinus and superior orbital fissure	+	+(< 10 %)	Blindness	Recurrence	154
	Proptosis; Blindness	L	Transfronto-orbital approach	Inside the muscle cone; lateral wall of the orbit; cavernous sinus and superior orbital fissure	+	+(< 10 %)	Improvement of the proptosis	None recurrence	43
11/M	Proptosis; Vision loss	L	Transfronto-orbital approach	Inside the muscle cone	+	+(< 5 %)	Improvement of the proptosis	None recurrence	57
12/F	Proptosis; Vision loss	L	Transfronto-orbital approach	Inside the muscle cone	+	+(< 5 %)	Improvement of the proptosis	None recurrence	74
13/F	Proptosis; Vision loss	R	Transorbital approach	Inside the muscle cone	+	+(< 10 %)	Improvement of the proptosis	Recurrence	36
	Proptosis; Vision loss; pain	R	Transfronto-orbital approach	Inside the muscle cone; lateral wall of the orbit; cavernous sinus and superior orbital fissure	+	+(< 10 %)	Improvement of the proptosis	None recurrence	125
14/M	Proptosis; Vision loss	L	Transfronto-orbital approach	Inside the muscle cone; lateral wall of the orbit; cavernous sinus	+	+(< 10 %)	Improvement of the proptosis	None recurrence	11
15/F	Proptosis; Vision loss	L	Transorbital approach	Inside the muscle cone;	+	+(< 10 %)	Improvement of the proptosis and vision	Recurrence	78
	Proptosis; Vision loss	L	Transfronto-orbital approach	Inside the muscle cone; lateral wall of the orbit; cavernous sinus	+	+(< 10 %)	Improvement of the proptosis	None recurrence	30
16/M	Proptosis	L	Transorbital approach	Outside the muscle cone lateral wall of the orbit	+	+(< 5 %)	Improvement of the proptosis	None recurrence	28

### Typical cases analysis

**Fig. 1:**
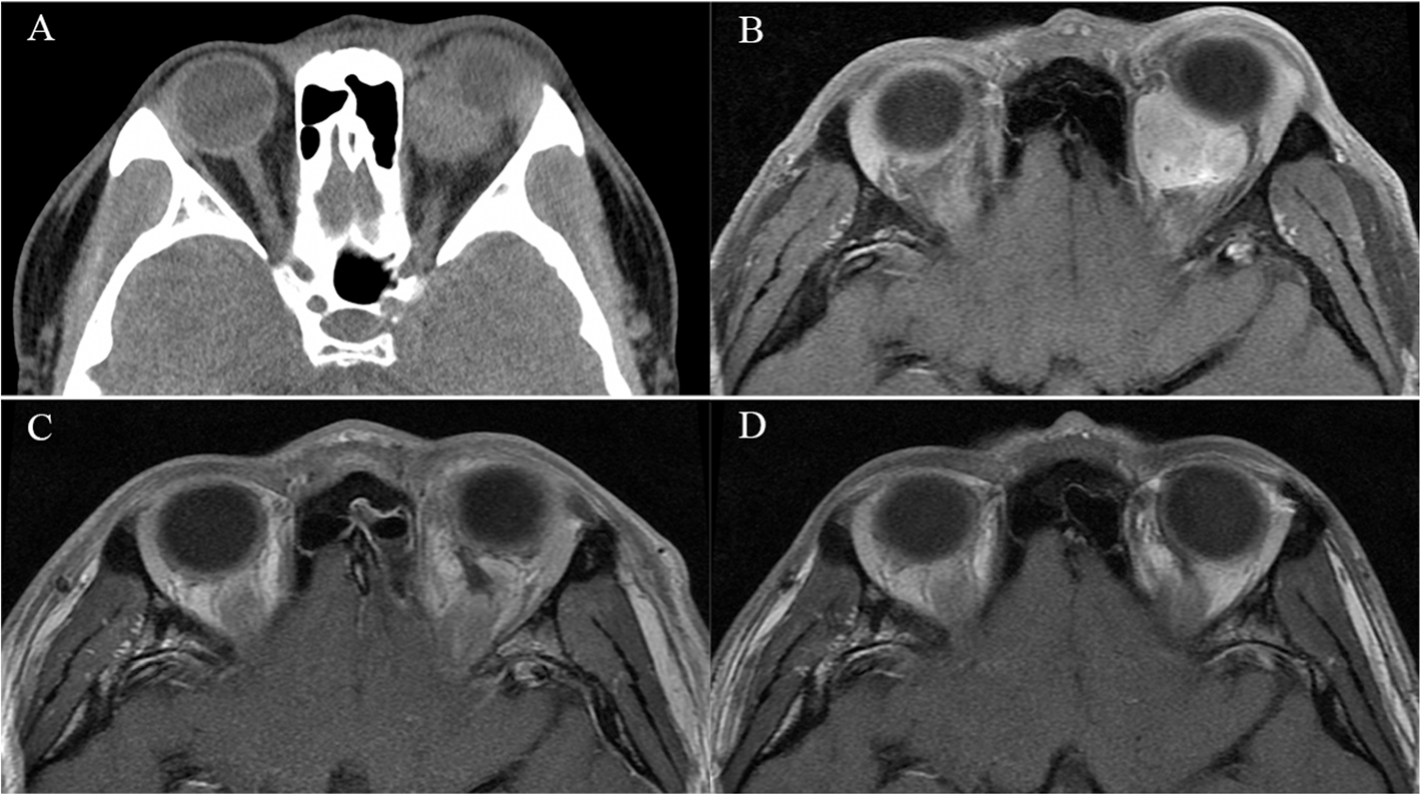
Case 1. The patient was a 23-year-old with a chronic disease process and left exophthalmos for more than 10 months. **a**, **b**: CT and MRI showed that the tumor was located in the muscle cone behind the eyeball and compressed the optic nerve. **c**, **d**: After the Transfronto-orbital approach was given to the craniotomy, the MRI re-examinations immediately after the operation and 1 year after the operation showed that the tumor was removed completely without recurrence, and the optic nerve was protected. There was no recurrence after 5 years of MRI follow-up

**Fig. 2:**
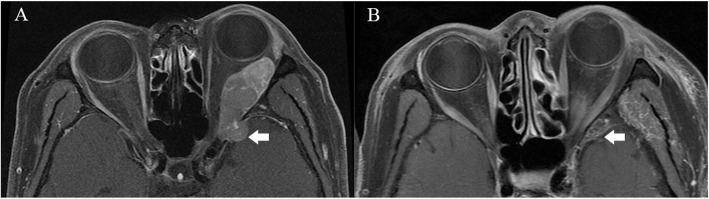
Case 2. The patient was a 45-year-old with a chronic disease process, left exophthalmos, and vision loss for more than 2 years. **a**: MRI showed that the main body of the tumor was located lateral orbit and invaded the intracranial dura through the superior orbital fissure (arrow). **b**: After the Transfronto-orbital approach was given to the craniotomy, the MRI re-examinations immediately after the operation showed that the tumor and involved dura were removed completely (arrow). There was no recurrence after 8 years of MRI follow-up

**Fig. 3:**
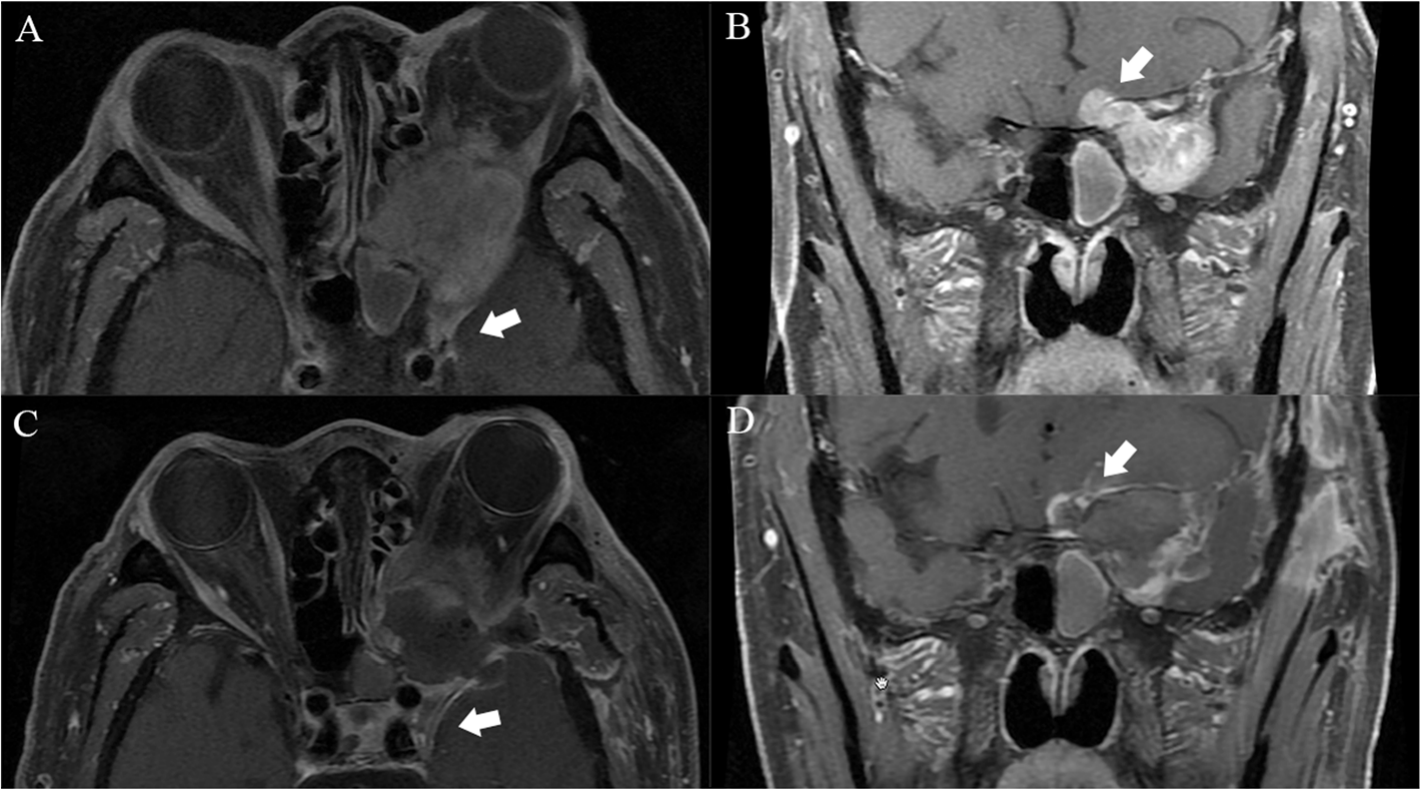
Case 3. The patient was a 78-year-old with a chronic disease process, left exophthalmos, and vision loss for more than 19 years. **a**: MRI showed that the main body of the tumor was located lateral orbit and invaded the intracranial dura, and cavernous sinus through the superior orbital fissure (arrow). **b**: The tumor broke through the dura mater of the skull base and invaded the intracranial structure (arrow). **c**, **d**: After the Transfronto-orbital approach was given to the craniotomy, the MRI re-examinations immediately after the operation showed that the tumors involving the cavernous sinus and intracranial were removed completely. There was no recurrence after 3 years of MRI follow-up

**Fig. 4:**
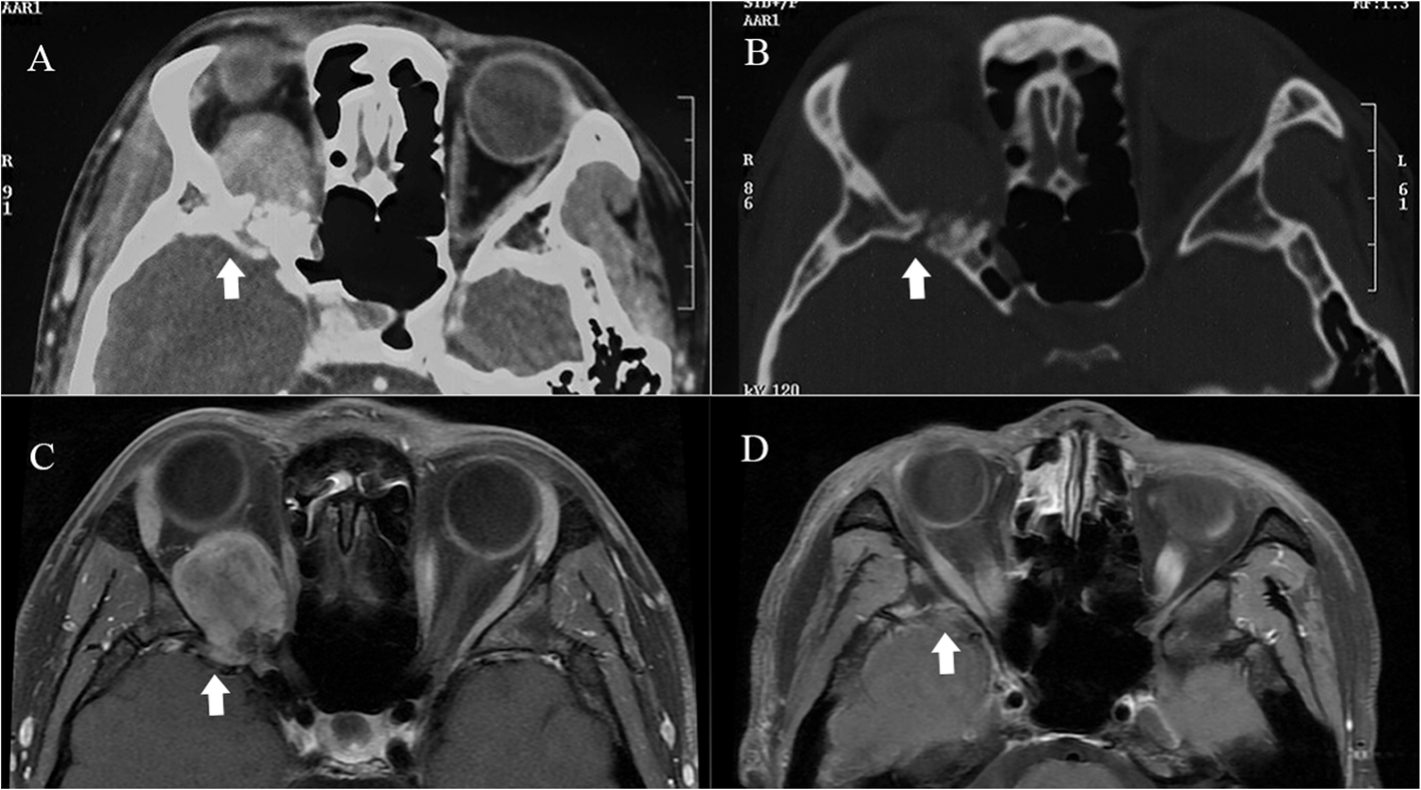
Case 4. The patient was a 25-year-old with chronic disease process and right exophthalmos for more than 6 months. **a, b**: CT showed that the tumor involved the bone of the lateral orbital wall with bone destruction (arrow). **c**: MRI showed that the main body of the tumor was located lateral orbit and invaded the intracranial dura through the superior orbital fissure (arrow). **d**: After the Transfronto-orbital approach was given to the craniotomy, the MRI re-examinations immediately after the operation showed that the tumors were removed completely. There was no recurrence after 7 years of MRI follow-up

**Fig. 5:**
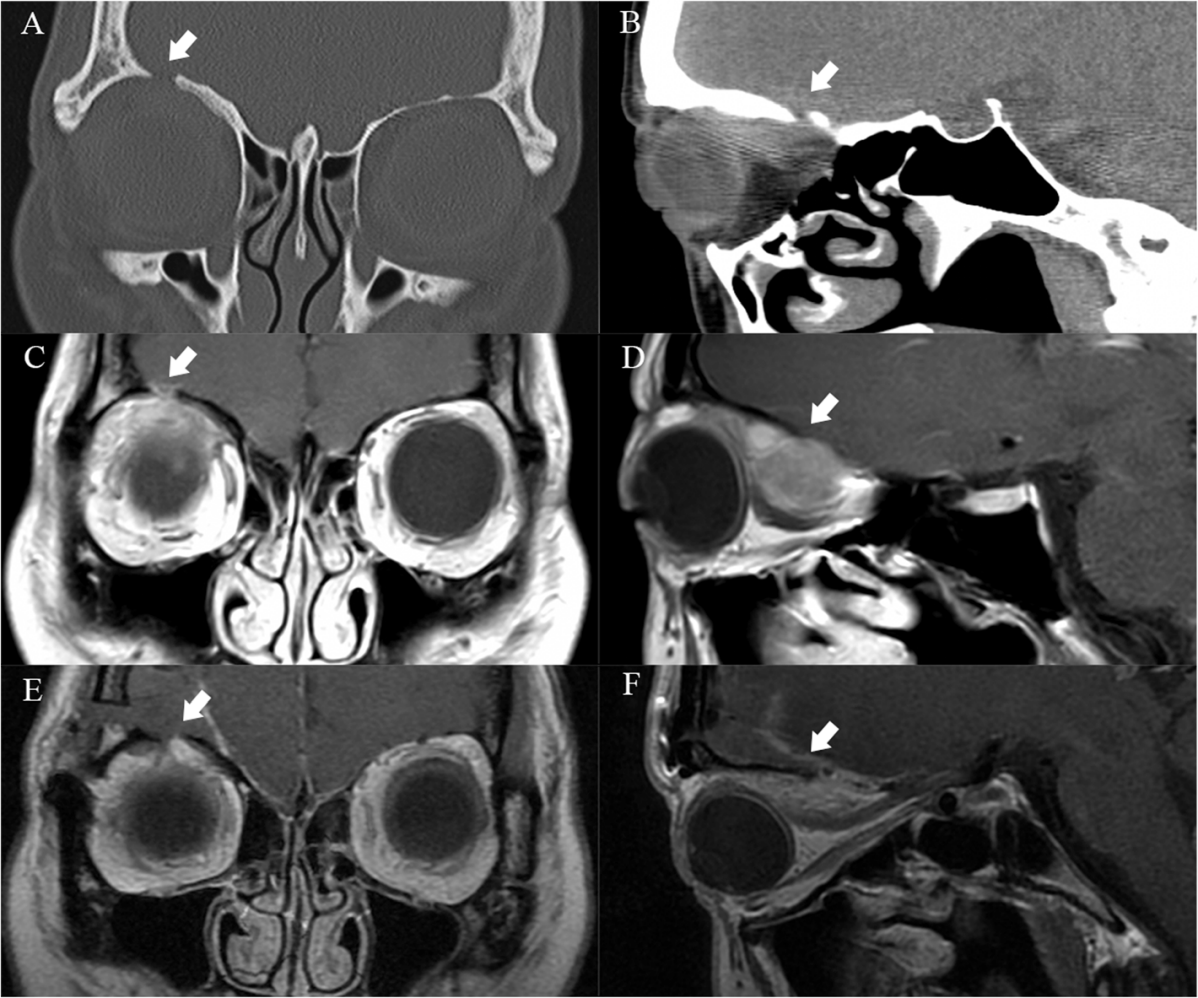
Case 5. The patient was a 46-year-old with a chronic disease process and right exophthalmos for more than 3 months. The patients underwent 2 operations (Transorbital approach) 12 years ago and 6 years ago. **a, b**: CT showed that the tumor involved the bone of supraorbital bone with bone destruction (arrow). C, D: MRI showed that the main body of the tumor invaded the intracranial dura through the destruction of the skull base bone (arrow). **e, f**: After the Transfronto-orbital approach was given to the craniotomy, the MRI re-examinations immediately after the operation showed that the tumors were removed completely. There was no recurrence after 5 years of MRI follow-up

**Fig. 6:**
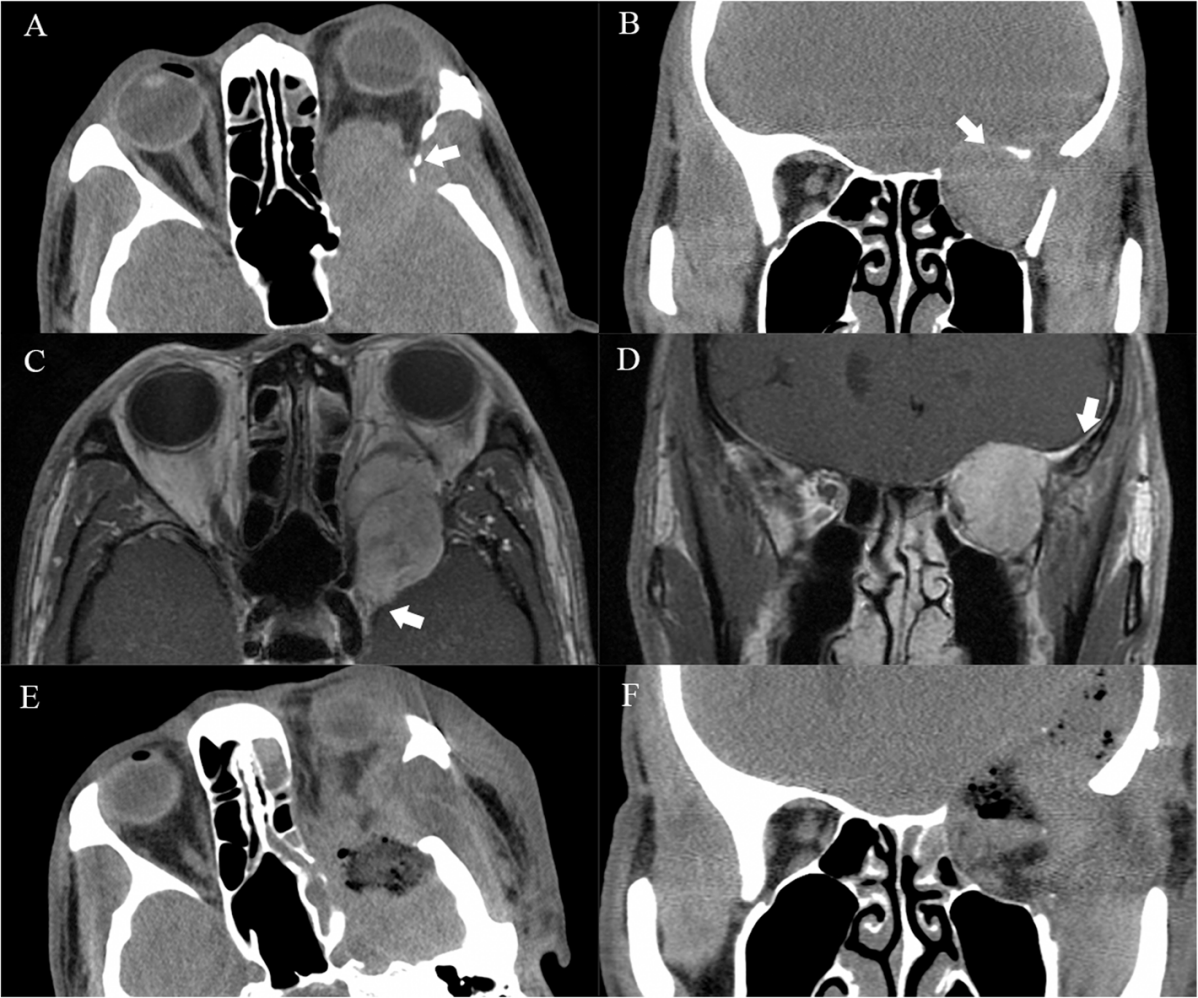
Case 6. The patient was a 48-year-old with a chronic disease process and left exophthalmos for more than 10 months. The patient underwent an operation (Transfronto-orbital approach) 6 years ago. **a, b**: CT showed that the tumor involved the bone of supraorbital bone with bone destruction (arrow). **c, d**: MRI showed that the main body of the tumor invaded the intracranial dura and cavernous sinus through the destruction of the skull base bone (arrow). **e, f**: After the Transfronto-orbital approach was given to the craniotomy, the MRI re-examinations immediately after the operation showed that the tumors were removed completely. There was no recurrence after 4 years of MRI follow-up

OSFT is an extremely rare intraorbital tumor. This tumor mostly originates from mesenchymal cells in the orbit and often involves the bone in the orbit. Even more, it breaks through the bony fissure of the cranial orbit and invades the intracranial structure. Most of these tumors require surgical treatment, and radiotherapy and chemotherapy are not sensitive. Clinically, the postoperative recurrence of tumors is very common. Most of these tumors are first diagnosed in ophthalmology, and most of the first operations are performed by ophthalmologists. However, some OSFTs are not suitable for ophthalmic surgery based on their location and scope of involvement. For example, the tumor is located in the retrobulbar muscle cone (Fig. 1). It is difficult to ensure the protection of the optic nerve through the ophthalmic surgical approach. Neurosurgery can better expose the tumor, give the incision of the periosteum of orbit, explore the medial side of the superior rectus muscle, and then completely remove the tumor. Sometimes OSFT grows into the skull along the supraorbital fissure and involves the dura mater. The lateral orbital approach is difficult to completely remove the tumor and at the same time, expand the bone and dura mater involved by the tumor. The remaining tumor will cause the tumor to recur. The craniotomy can fully expose the supraorbital tumor. After the transfronto-orbital approach was given to the craniotomy, the MRI re-examinations immediately after the operation showed that the tumor and involved dura were removed completely (Fig. 2). When the tumor further involves the intracranial structure, it will invade the cavernous sinus and even the intracranial brain tissue (Fig. 3). In this situation, craniotomy can still completely remove the tumor. Generally, it is difficult for the tumor to break through the bilateral dural structure of the cavernous sinus. Adequate exposure, remove the tumor in pieces, and protect the internal carotid artery, oculomotor nerve, trochlear nerve, abductor nerve, and trigeminal nerve (V1, V2). The enlarged surgical field can not only prevent the traction of the orbital contents but also ensure that all the tumor-involved areas are removed. In our clinical cases, we found that most of the OSFT involved periorbital bone to varying degrees, and all craniotomy operations involved extensive removal of the involved bone (Figs. 4, 5 and 6). The tumors located on the lateral or superior side of the orbit are often accompanied by bone destruction, absorption, and hyperplasia. Wrong surgical procedures and missed imaging studies often accompany tumor recurrence. The forcible removal of the skull base through the transorbital approach may result in cerebrospinal fluid leakage contusion and laceration of the brain. It is difficult to adequately deal with the involved periorbital or skull base bone with the ophthalmic surgical approach, which left a hidden danger to the recurrence of the tumor. In the case of tumors involving the periorbital and skull base bones, we have adopted craniotomy. The tumor is completely resected through the transfronto-orbital approach. It is worth noting that during the operation, the affected bone must be extensively removed, and the involved dura mater and brain tissue must be removed completely. In this study, most of the patients we treated were patients who relapsed after ophthalmic surgery or patients who were difficult to remove completely with ophthalmic surgery alone. For this type of patient, we believe that the can give a total resection of the tumor and reduce the recurrence of the tumor.

## Discussion

The outcomes of this study presented that sixteen patients with OSFT underwent a total of 29 operations, of which 12 were transorbital approach operations and 17 were transfronto-orbital approach operations. Ten patients (10/16, 62.5 %) had recurrence. The recurrence rate of transorbital approach operations was 83.3 % (10/12), and the recurrence rate of transfronto-orbital approach operations was 17.6 % (3/17). No patients had treatment-related complications.

**Fig. 7:**
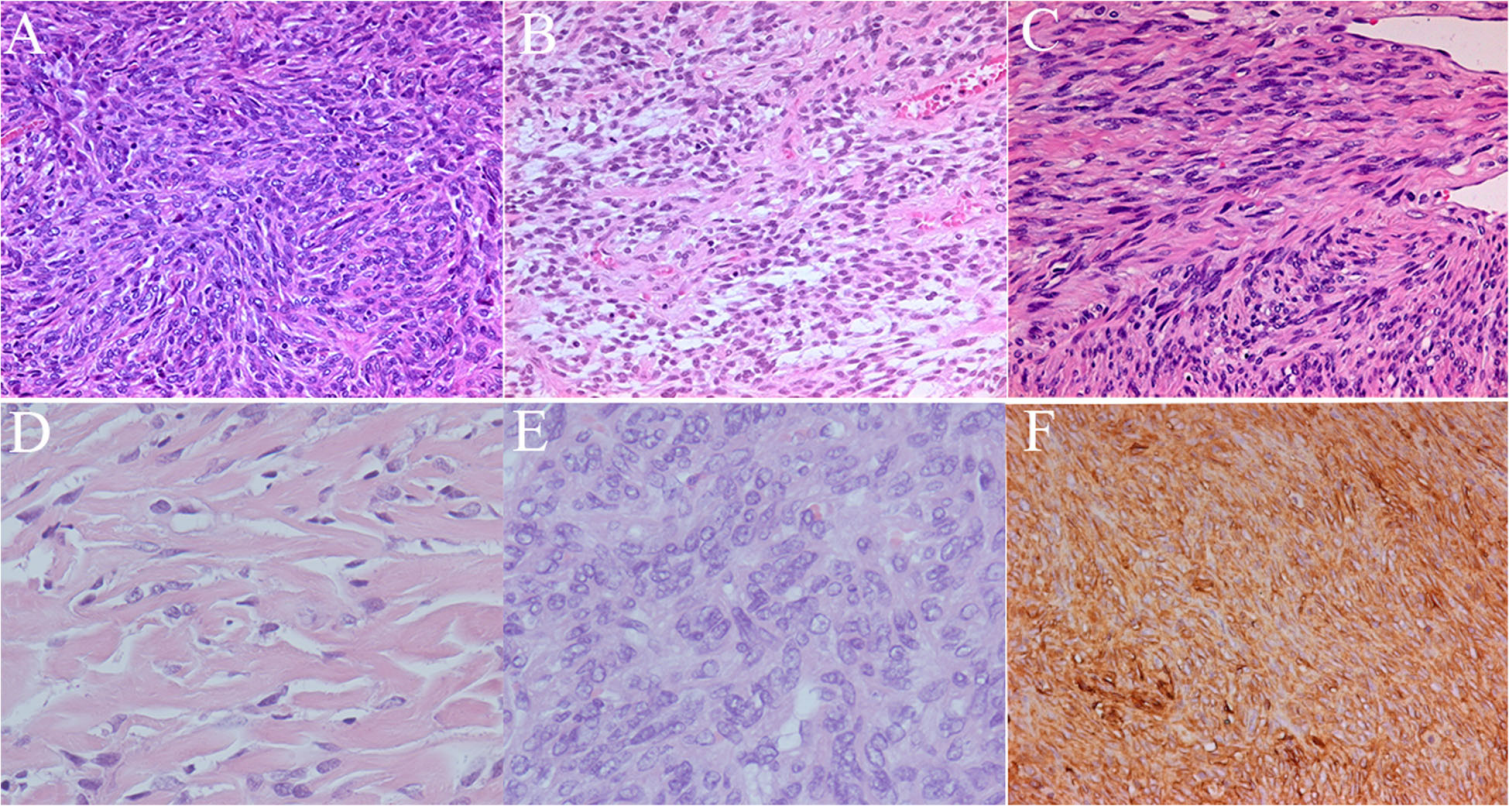
**a, b, c**: The pathological pictures of 3 patients with recurrent tumors after multiple operations showed short fusiform tumor cell proliferation with more collagen and blood vessels between the cells (HE 200). **d, e**: The pathological pictures of 2 patients without tumor recurrence after one operation showed that the short spindle tumor cells proliferated with a small amount of collagen and vascular components among the cells (HE 200). **f**: All the tumor specimens showed CD34 cytoplasmic positive

SFTs grow slowly and rarely encounter mesenchymal tumors. They are composed of spindle-shaped cells and are known to mainly affect the pleura. Most OSFTs are described in isolated case reports; thus their clinical behavior is currently unknown. It is generally believed that OSFTs behave in a benign fashion and follow a non-aggressive course. A few of the OSFT cases reported have displayed malignant histological features [18, 19]. Immunohistochemically, the expression of CD34 may be lost in tumors that undergo malignant transformation in cases of OSFTs. However, these pathologic factors do not always correlate with the clinical behavior of the tumor. The CD34 was positive in all 29 pathological samples in our research, whether the tumor recurred or not. Importantly, Furusato et al. reported that p53 and Ki-67 have been associated with worse outcomes and higher mitotic index [20]. In our study, when the tumor was in borderline or malignant tendency, Ki-67 index was 10 % − 20 %, but there was no significant correlation between tumor recurrence and Ki67 index. The Ki-67 index of all 29 surgical samples were 5 % − 20 %, regardless of recurrence. In our research, tumor recurrence had no obvious correlation with pathology and immunohistochemistry (Fig. 7). The tendency of borderline or malignant tumors that we see in the clinic was just that the so-called cell number and vascular structure increase in pathology, and the cell morphology had not changed. At the same time, this type of tumor only showed that it was easy to recur in situ. In our database none of the cases had distant metastases. Compared with the pathological pictures of patients without tumor recurrence after one operation, the pathological pictures of patients with recurrent tumors after multiple operations showed short fusiform tumor cell proliferation with more collagen and blood vessels between the cells.

In the review subjects of our study, through up to 17 years of follow-up and careful reading of the pathology of 29 operations, we did not find the obvious differences between cytomorphology of tumors with potential malignancy or recurrence and tumors that had not recurred. However, most tumors with malignant transformation and recurrence tended to be accompanied by more short spindle-shaped tumor cell proliferation and intercellular collagen vessels. We believed that the recurrence of tumors mainly depends on the different surgical methods adopted for different tumor locations. We reviewed more than 20 years of literature and found that few ophthalmologists could osteotomy the bone involved in OSFTs. There were 2 main reasons. One was that the ophthalmologists underestimated the nature of the OSFTs and only performed in situ resection; the other was that they did not read the orbital CT and MRI carefully, ignoring that the tumor easily invaded the intracranial structure through the cranio-orbital junction. Only used a transorbital approach, leaving intracranial tumors. Many OSFTs will involve the bone of the orbital wall, and some tumors will further involve the intracranial structure through the superior orbital fissure. In clinical practice, the bones of the OSFTs involving the orbital wall will be removed as much as possible. Similarly, incomplete resection of the dura, cavernous sinus, and intracranial structures involved in the tumor will definitely lead to tumor recurrence. Few centers currently recognize the importance of OSFT to remove the affected bone [21]. Ophthalmologists must have a full understanding of OSFT and give CT and MRI screenings. CT and MRI are currently still important evaluation methods to judge the extent of the tumor and surrounding tissue involvement [22, 23]. Read carefully the impact of the tumor on the bone quality of the orbital wall, and do not miss the clues that the tumor is involved in the intracranial structure and adopt the wrong surgical procedure. OSFT is a kind of tumor with the tendency to become cranio-orbital tumor. We advocate multidisciplinary cooperative surgery. Neurosurgery combined with ophthalmic surgery can not only give better preoperative evaluation and selection of surgical approaches but also greatly improve the incidence of postoperative complications.

OSFTs are so rare that most published cases were individual case reports without documented extended follow-up; therefore, it is difficult to predict the true clinical course of these tumors. In our study, we followed up the clinical data of 16 patients for 17 years. We hope that in our research data, we will give our center’s analysis opinions on the clinical features and surgical outcomes of such tumors. As a neurosurgery department, most of the 16 cases of OSFT admitted to our department had some neurosurgery indications in common. In 13 patients, the tumors involved the lateral orbital wall of the greater wing of the sphenoid bone, the cavernous sinus, and supraorbital fissure were involved in 11 patients, and the intracranial structures were involved in 4 patients. When the tumor involved the pterygoid bone, the bone was usually accompanied by hyperplasia, absorption, or destruction. Although the tumor can be surgically removed through the lateral orbital approach, a part of the lateral orbital part of the greater wing of the sphenoid bone was adjacent to the intracranial dura mater and cavernous sinus structure. It is difficult for ophthalmologists to completely deal with the involved bone and complete resection of the bone, which leaves a sequela for the recurrence of the tumor. When the tumor is located in the muscle cone and grows into the intracranial through the supraorbital fissure and adjacent to the cavernous sinus structure, it is more difficult to completely remove the tumor by ophthalmic surgery, and the risk of surgery is also increased accordingly. In addition, when the tumor is located in the muscle cone and located behind the eyeball and adjacent to the intra-orbital structures such as the optic nerve, it is difficult to completely expose and remove the tumor through the transorbital approach.

Among the OSFT patients received by our department, the patients who underwent tumor resection through the lateral orbital approach did not have extra resection of the lateral orbital wall bone, and the recurrence rate of the operation reached 83.3 %. When the tumor recurred through the transorbital approach, involving the cavernous sinus supraorbital fissure, or located in the posterior ocular muscle cone, we performed craniotomy through the transfronto-orbital approach. We routinely removed the frontotemporal bone flap and the eyebrow arch bone to increase the exposure of orbital contents, the lateral wall of the cavernous sinus, and the whole wing of the sphenoid bone. The involved orbital bone, cavernous sinus tumors, dura mater, and even intracranial tumors were completely removed. The postoperative recurrence rate was controlled at 17.6 %. It should be emphasized that OSFT is a potentially malignant tumor, and total tumor resection is essential for tumor recurrence. It can be seen that even with different surgical approaches, tumors still have a high postoperative recurrence rate, which may be related to the pathological characteristics of OSFT, so long-term follow-up of tumors is very important. The treatment of choice is complete surgical resection with a long-term follow-up [24]. Recurrence after surgery has been reported [25]. Incomplete surgical resection is probably the most important cause of recurrence [16]. The prognosis of the patient depends on several factors, one is the nature of the disease and whether it is malignant or benign because the prognosis of the malignant form has a worse outcome [26]. Orbital tumors tend to show a higher frequency of local recurrence than distant metastasis [27]. No distant metastasis occurred in our cases. Whatever the management plan, clinical follow up is essential for any patient.

Regarding the postoperative recurrence rate of OSFT, each center has different results because of the nature, the location, the operation method, and the first clinic of the tumors [28]. In recent years, some studies have pointed out that immunohistochemical analysis showed that not only CD34 and Bcl2 are positive in tumor cells, but also nuclear STAT6 is positive, indicating that the tumor may be a rare variant of SFT. But STAT6 cannot predict tumor recurrence after surgery. We cannot predict the biological behavior of tumors through STAT6. Initial surgical resection should be complete in order to avoid recurrence [29–31].

### Limitations

There were several limitations in this study. Firstly, this trial was not a randomized controlled trial. Secondly, this study was only single-center trial and the sample size was limited. Thirdly, the clinical follow-up was short and it was necessary to observe the clinical long term prognosis.

## Conclusions

OSFT is an extremely rare tumor. The main pathological feature of this tumor is a benign tumor, and the cell morphology of the tumor with multiple recurrences and malignant growth trend has not changed in the pathological and immunohistochemical samples. OSFT has a tendency to grow toward the cranio-orbital junction. The postoperative recurrence rate of OSFT is relatively high, so complete tumor resection is very important for prognosis. Inappropriate surgical approaches can lead to incomplete removal of the tumor and cause recurrence. Choosing the correct operation approach according to the position of the OSFT in the orbit and complete removal of the dura mater and bone affected by the tumor is crucial for the prognosis. Nevertheless, regular long-term follow-up after complete resection is necessary.

## Data Availability

The datasets used and analysed during the current study available from the corresponding author on reasonable request.

## Abbreviations

OSFT: Orbital solitary fibrous tumor
SFT: Solitary fibrous tumor
IQR: Interquartile range
